# Limiting factors for queen conch (*Lobatus gigas*) reproduction: A simulation-based evaluation

**DOI:** 10.1371/journal.pone.0251219

**Published:** 2022-03-09

**Authors:** Nicholas A. Farmer, Jennifer C. Doerr

**Affiliations:** 1 NOAA Fisheries Southeast Regional Office, St. Petersburg, Florida, United States of America; 2 NOAA Fisheries Southeast Fisheries Science Center, Galveston, Texas, United States of America; Ocean Frontier Institute, CANADA

## Abstract

Queen conch are among the most economically, socially, and culturally important fishery resources in the Caribbean. Despite a multitude of fisheries management measures enacted across the region, populations are depleted and failing to recover. It is believed that queen conch are highly susceptible to depensatory processes, impacting reproductive success and contributing to the lack of recovery. We developed a model of reproductive dynamics to evaluate how variations in biological factors such as population density, movement speeds, rest periods between mating events, scent tracking, visual perception of conspecifics, sexual facilitation, and barriers to movement affect reproductive success and overall reproductive output. We compared simulation results to empirical observations of mating and spawning frequencies from conch populations in the central Bahamas and Florida Keys. Our results confirm that low probability of mate finding associated with decreased population density is the primary driver behind observed breeding behavior in the field, but is insufficient to explain observed trends. Specifically, sexual facilitation coupled with differences in movement speeds and ability to perceive conspecifics may explain the observed lack of mating at low densities and differences between mating frequencies in the central Bahamas and Florida Keys, respectively. Our simulations suggest that effective management strategies for queen conch should aim to protect high-density reproductive aggregations and critical breeding habitats.

## Introduction

The queen conch (*Lobatus gigas*, formerly *Strombus gigas*) occurs throughout the Caribbean Sea, in the Florida Keys and Gulf of Mexico, and around Bermuda. They are a mollusk characterized by a large, heavy, whorl-shaped shell with multiple short spines at the apex, a brown and horny operculum, and a pink interior of the shell lip [1]. Shell morphology is influenced by environmental conditions including habitat [2, 3]. Females are generally slightly larger than males [4]. Queen conch are benthic-grazing herbivores that feed on diatoms, seagrass detritus, and various types of algae and epiphytes [5, 6]. Adult distributions are heavily influenced by food availability and fishing pressure; in unexploited areas, they are most common in shallow marine waters less than 30 m depth [3]. Adults prefer sandy algal flats, but are also found on gravel, coral rubble, smooth hard coral, and beach rock bottoms [7–9].

Adult conch have a protracted spawning season of 4–9 months, with peak spawning during warmer months [1, 10, 11]. They reproduce through internal fertilization, meaning individuals must be in contact to mate. Copulation has been documented day and night [1]. Females can store fertilized eggs for several weeks [10], and egg masses may be fertilized by multiple males [12]. Egg laying takes 24–36 hours, with each egg mass containing about 750,000 eggs [13]. Fecundity appears to be influenced by food availability; with adequate food, females lay an average of 13.6 egg masses during a single reproductive season, compared to an average of 6.7 egg masses containing 500,000 eggs each when food is limited [13].

Queen conch are relatively slow moving, averaging only a few meters of movement per day [14–16]. The average home range size for an individual queen conch is variable and has been measured at 5.98 ha in Florida [14], 0.1 to 1.85 ha in Mexico [16], 0.6 to 1.2 ha in Barbados [17], and 0.15 to 0.5 ha in the Turks and Caicos Islands [18]. Movement rates increase and are fastest in the summer, possibly due to warmer waters promoting increased metabolic activity as well as increased movement related to mate seeking during the reproductive season [14]. In many locations, adult conch migrate to different habitat types during their reproductive season, and then return to feeding grounds [5, 14, 16, 19]. Geographically isolated conch in some areas of Florida and Puerto Rico remain in deep water year-round [14, 20].

Queen conch are among the most economically, socially, and culturally important fishery resources in the Caribbean [21, 22], with high domestic and exported landings [23]. Queen conch are listed in CITES Appendix II, requiring non-detriment findings to allow for export quotas. The fishery consists of both industrial and artisanal fleets and encompasses the entire Caribbean. Commercial exports increased in the 1980s and 1990s, with a peak of around 3000 tonnes in 1996 and 1997 [23]. These increased landings were accompanied by decreasing population densities across the range [24, 25]. Management approaches vary across the region, but include size restrictions, closed seasons, harvest quotas, and/or gear restrictions. Despite these management interventions, many populations have not recovered [26–28]. In the United States, overharvesting and habitat loss precipitated the collapse of large commercial and recreational fisheries in south Florida. Despite closure of the commercial fishery in 1976, followed by closure of the recreational fishery in 1986, the population has not recovered [29, 30]. NOAA Fisheries is currently engaged in an Endangered Species Act status review for queen conch to evaluate threats to the species’ habitat, overutilization, and the adequacy of existing regulatory mechanisms to protect the species from extinction [31].

Empirical observations have suggested mating and egg laying in queen conch are directly related to the density of mature adults [32–34]. In animals that aggregate, low population densities can make it difficult or impossible to find a mate [32, 35–37], an issue which is likely compounded for slow-moving animals such as conch [14, 15]. Observations of queen conch populations also suggest an Allee effect, where little to no mating occurs below a critical density threshold [32, 34, 38].

In this study, we present a mechanistic simulation model of conch movement and reproductive behavior to evaluate whether issues with mate finding at low population densities are sufficient to explain empirical observations of mating frequency. We further evaluate the roles of movement speed, rest periods between mating events, perception of and/or attraction to conspecifics, sexual facilitation, and restricted movement upon mating success. This approach provides a stochastic, quantitative approach towards evaluating the relative contributions of a myriad of factors to individual reproductive success and overall population reproductive output. Comparing emergent properties [39] from individual-based mechanistic simulations to empirical observations can benefit hypothesis elimination and facilitate identification of the biological processes driving queen conch reproductive success.

## Materials and methods

### Model configuration

To test whether challenges in mate finding at low densities could explain mating frequency patterns observed in the field, male and female conch movements, mating (i.e., collisions), and spawning (i.e., egg laying) were simulated in R [40] using package ‘*particles’* [41] (S1 File). The simulation assumes a constant unit time step *Δt* = 1 d for each step and a constant unit mass *m* = 1 for all particles (i.e., adult conch). As a result, a force *F* acting on a conch is equivalent to a constant acceleration *a* over the time interval *Δt* and can be simulated simply by adding to the conch’s velocity, which is then added to the conch’s position.

**Fig 1:**
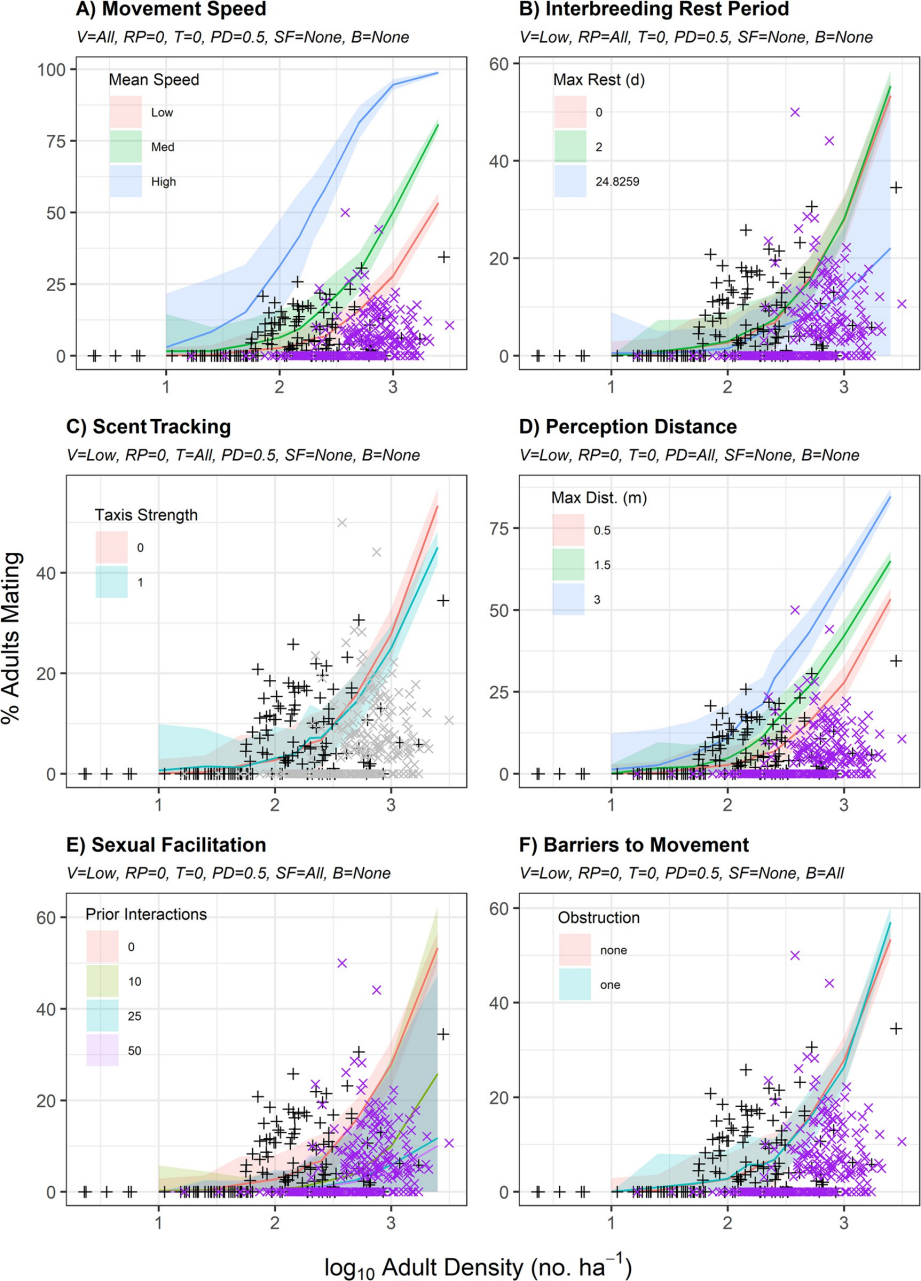
Percent mating relative to model parameters. Mean (solid lines) and 95% confidence band (shaded ribbons) for percent of simulated adult queen conch successfully mating relative to log_10_ adult density (No./ha) relative to variation in A) movement speed (V), B) interbreeding rest period (RP), C) scent tracking (T), D) perception distance (PD), E) sexual facilitation (SF), and F) barriers to movement (B), with all other variables fixed at their lowest simulated values. Empirical observations by Stoner & Ray-Culp [32] and Stoner et al. [34] (black crosses) and Delgado & Glazer [38] (purple x’s) are overlaid for comparison.

Sexually mature adult conch were randomly distributed at specified densities (i.e., 10 to 2500 adults/ha) on a 1-ha grid using the *aquarium_genesis* function [41] and tracked over one-day time steps (Fig 1). The range of simulated densities was selected to provide meaningful contrast and contained the mean densities reported by Stoner et al. (209 conch/ha) [34], Delgado & Glazer (610 conch/ha) [38], and Stoner et al. (2,293 conch/ha) [34], but not the maxima reported by Delgado & Glazer (3,133 conch/ha) [38]. Daily reproductive dynamics were simulated across 10 days within peak spawning season [1, 10, 11], generating 10 replicates of daily random movements across a specified number of individuals per run. Movement velocities were randomized each step using the *random_force* function [41], corresponding to a simple isotropic random walk model (SRW) [42]. This force applies a random velocity modification to all particles. The model assumes no directional persistence between daily time steps, following reported field observations [18, 43]. The modification is uniformly distributed and bound by the parameters provided during initialization. The number and frequency of mating and spawning events was tracked and averaged across individuals over these 10 replicates. To evaluate whether mechanistic processes might provide superior fits to empirical observations over changes in density alone, simulation scenarios evaluated the additional influence of movement speed, scent tracking, interbreeding rest period, sexual facilitation, and conspecific perception distance (Table 1). All combinations of variables were tested using custom written R software (S1 File), with 43,200 bootstrap simulation runs representing 432,000 spawning days across 20,671,200 individuals.

**Table 1:** Queen conch reproductive parameters.

Parameter	Symbol	Units	Value	Source(s)
Adult Density	N	N ha^-1^	10, 25, 50, 100, 150, 200, 250, 500, 1000, 2500	Stoner et al. [34], Delgado & Glazer [38]
Movement Speed	V	M d^-1^	Low: N^a^ [2.57 ± 1.57 (1.47–3.56)] Med: N^a^ [4.17 ± 0.41 (0–5.52)] High: N^a^ [11.36 ± 0.24 (0–21.24)]	Glazer et al. [14], Doerr & Hill [15]
Interbreeding Rest Period	RP	d	0 U^b^ (0–2) N^a^ [8.7 ± 4.9 (0–24.83)]	Weil & Laughlin [11]
Scent Tracking	T	n/a	0 1 (22 m max taxis distance)	Davies & Blackwell [44], Ng et al. [45], Doerr & Hill [15]
Perception Distance	PD	m	U^b^ (0,0.5)	Informal expert elicitation from A. Stoner, G. Delgado, R. Glazer
			U^b^ (0,1.5)	
			U^b^ (0,3.0)	
Sexual Facilitation	SF	# prior interactions	0, 5, 10, 25, 50	Appeldoorn [35], Gascoigne & Lipcius [57], Delgado & Glazer [38]
Barriers to Movement	B	n/a	none, one	n/a

Table showing the parameters evaluated in simulation models.

^a^Truncated normal distribution [Mean ± SD (Min–Max)]

^b^Uniform distribution

### Movement speed

Daily movement speeds have been estimated through acoustic telemetry [14, 15]. Adults move at varying speeds throughout the year with movement rates increasing during seasonal migrations and slowing during foraging activities or upon reaching mating aggregations. We selected a low speed scenario from male movement speeds and a medium speed scenario from summer movement speeds reported by Glazer et al. [14]. We selected a high movement speed scenario from pre-aggregation migratory movements reported by Doerr & Hill [15]. Movement speeds were randomly generated from a truncated normal distribution (Table 1) for each day and individual. Movement directions were random, drawn from a uniform distribution from 0 to 360 degrees, unless animals encountered a barrier or scent tracking was enabled. Movement directions were random because the time scale was set to 1 day and animals were assumed to be at a spawning aggregation engaged primarily in the search for potential mates.

### Interbreeding rest period

Female receptivity to mating was evaluated as a possible interbreeding ‘rest period’ between mating events (Table 1). Successful spawning events were counted when a female with a recorded mating event during the spawning season deposited an egg mass. Time required for oogenesis between spawning events were parameterized based on observations by Weil & Laughlin [11]. Because females can store viable sperm from a single copulation for several weeks [11, 44], no additional timing requirements were imposed for spawning beyond one prior mating event during the spawning season.

### Scent tracking

Scent tracking has been postulated as an energy-saving mechanism in gastropods [45, 46]. Because queen conch do not move using a slime trail, it is unclear if scent tracking is possible for the species. If queen conch do chemically track conspecifics, it might be accomplished through sex hormones [47]. For most simulations, scent tracking was disabled. When enabled, scent tracking was modeled as particle attraction using the *manybody_force* function at a specified level of taxis distance and strength [41]. This force implements an *n*-body simulation using the Barnes-Hut approximation for improved performance; this approximation replaces a group of distant points with their center of mass. In exchange for a small amount of error, this scheme significantly speeds up calculation, with complexity *n log n* rather than *n*^2^, where *n* is the number of nodes. An *n*-body simulation calculates attraction between all particles in a system based on their relative distances and the strength of attraction. For each application, a quadtree stores the current node positions; then, for each node, the combined force of all other nodes on the given node is computed. For a cluster of nodes that is far away, the charge force can be approximated by treating the cluster as a single, larger node. The theta parameter, set at the default of 0.9, determines the accuracy of the approximation: if the ratio w / l of the width w of the quadtree cell to the distance l from the node to the cell’s center of mass is less than theta, all nodes in the given cell are treated as a single node rather than individually.

When enabled, the distance of influence and duration of scent tracking were constrained to 22 m and 1 day, respectively. Specifying a maximum distance improves computational performance, creates a more biologically realistic taxis process, and produces a more localized layout. The taxis distance constraint was set based on the maximum movement speed (Table 1) and the duration constraint was set based on the time step of the simulation. These constraints were intended to reflect the pragmatic constraints of scent trail decay in a dynamic marine environment, as the actual perception distance and duration of scent trails is unknown, but unlikely to exceed these constraints. Scent tracking was assumed to be omnidirectional over the one-day time step. Although this assumption was driven by computational simplicity, ecologically it could be interpreted as changes in tidal- or wind-driven current patterns or benthic deposition of hormones during random movements within the discrete one-day time step.

### Perception distance

A successful mating event was counted when a male encountered a receptive female. An encounter was defined as the daily paths of two individuals being within the randomly selected perception distance for those individuals. Conspecific perception distance is unknown for queen conch; thus, an informal expert elicitation process was used to parameterize this variable (Table 1). This expert elicitation involved informal interviews with three experts in the field and was resolved into a range of reasonable values. Simulated variability was intended to capture differences in visibility, benthic habitat, and water currents that might carry scent-based cues.

### Sexual facilitation

Sexual facilitation has been postulated as an additional mechanism whereby total reproductive output increases with density as rates of gametogenesis and spawning increase due to stimulation by members of the opposite sex [35, 48–50]. Sexual facilitation was modeled as a positive feedback loop between direct contact or perception of males through chemical cues [35] and receptivity to mating in females [35, 48–50]. Sexual facilitation was modeled as a stochastic process where the likelihood of a female *i* successfully mating at time *t* increased linearly with the number of prior contacts (*C*) with males, up to threshold τ, where mating would be 100% successful:

$${Mating}_{t}\to \left(U(0,1)<(\sum_{t=1}^{t}C_{i}/\tau )\right)$$ (1)

Although sexual facilitation has been demonstrated in other prosobranchs [51–54], it has not been empirically demonstrated in queen conch. Similarly, the accelerated rate of gametogenesis conferred through sexual facilitation is unknown. To encompass these uncertainties, sexual facilitation was run with τ = 0, 5, 10, 25, and 50, respectively, where τ = 0 represents no sexual facilitation required.

### Barriers to movement

A movement barrier was simulated to evaluate the impacts of microhabitat features on reproductive dynamics. A single linear barrier was centered in the one-hectare simulation arena, restricting connectivity between the two sides of the arena by 80% (S1B Fig in S1 Fig). If a conch’s daily random movements took it into the barrier, it was automatically moved to the closest point at the exterior of the barrier. In the subsequent time step, it would randomly move from that point, drawing from the movement speed distribution and a random movement direction between 0 to 360 degrees. Other barriers were developed in the model code but not explored in depth for this study due to computational time requirements (S1 Fig).

### Comparisons to empirical data

Percent mating and percent spawning were compared to data from the central Bahamas [32, 34] and the Florida Keys [38]. The data from Delgado & Glazer [38] were filtered to May-July only, to mirror the “peak spawning” season simulated in the model (see Fig 4 in Delgado & Glazer [38]). Logistic regression models were fit to percent mating versus log density from field observations in the central Bahamas [32, 34] and Florida Keys [38] using R package ‘*drc’* with the lower asymptote fixed at zero [55]. Non-mating events from simulations run with densities >100 adults/ha were excluded from logistic regression models since this number represents the critical threshold above which there is an increased probability of successful mating. Observations from Delgado & Glazer [38] of no mating activity at >100 adults/ha could represent potential lag times before mating, that the aggregation was not yet in peak reproductive condition, or the presence of some other environmental factor influencing mating frequency within the empirical data. Data from Stoner & Ray-Culp [32] and Stoner et al. [34] were obtained from 213 aggregation surveys across four years (1995, 2009, 2010, and 2011). Of these, only Stoner & Ray-Culp [32] provided records of percent spawning. Data from Delgado & Glazer [38] were obtained from 341 aggregation surveys across 20 years, with records of percent mating and percent spawning. It is important to note that population densities from these studies were derived using different methodological techniques; Stoner & Ray-Culp [32] and Stoner et al. [34] conducted randomized shelf-wide transect surveys while Delgado & Glazer [38] conducted directed intra-aggregation transects. These datasets comprised the most extensive and best available reproductive frequency information with which to generate comparisons with our model simulations and subsequent interpretation was performed cautiously.

Point estimates for comparison with empirically-observed densities were generated from each bootstrap run of the simulation model through linear interpolation using the *approx* function in R package ‘*stats’* [56]. To evaluate the efficacy of the simulation model at capturing observed trends, goodness of fit was compared between simulation model interpolations and log-logistic (ED50 as parameter) regression fits to empirical data from the central Bahamas [32, 34] and Florida Keys [38] using R package ‘drc’ [55] with lower limit at 0. Goodness of fit was summarized as sum of squared residuals (SSR), mean square error (MSE), and root mean square error (RMSE). Goodness of fit between simulation-based extrapolation fits and empirical data-based regression fits was compared using Bonferroni-corrected paired t-tests, assuming unequal variances with a significance threshold of α < 0.05, to compare the residuals from both approaches.

## Results

**Fig 2:**
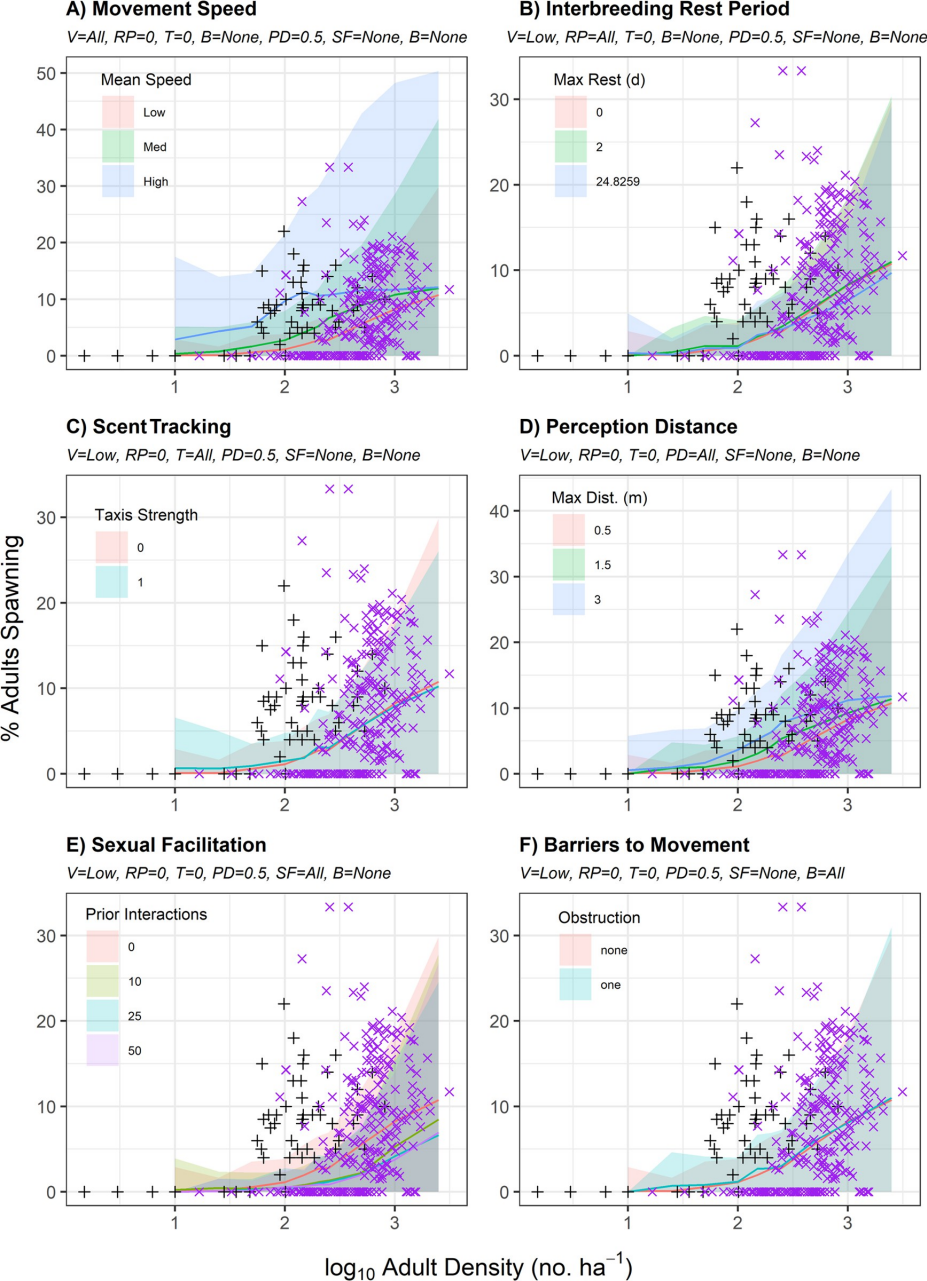
Percent spawning relative to model parameters. Mean (solid lines) and 95% confidence band (shaded ribbons) for percent of simulated adult queen conch spawning relative to log_10_ adult density (No./ha) relative to variation in A) movement speed (V), B) interbreeding rest period (RP), C) scent tracking (T), D) perception distance (PD), E) sexual facilitation (SF), and F) barriers to movement (B), with all other variables fixed at their lowest simulated values. Empirical observations by Stoner & Ray-Culp [32] and Stoner et al. [34] (black crosses) and Delgado & Glazer [38] (purple x’s) are overlaid for comparison.

The influence of density, movement speed, interbreeding rest period, scent tracking, conspecific perception distance, sexual facilitation, and barriers to movement on successful mating and spawning are presented in Figs 1 and 2, respectively. All simulation-based approaches overestimated mating activity, but not spawning activity, at high adult densities (>1000 adults/ha; Figs 1 and 2). Increased movement speed led to increased mating (Fig 1A) and increased spawning (Fig 2A), especially at low densities. The high movement speed scenario overestimated mating activity at all densities, and all movement speed scenarios over-predicted mating activity at densities of fewer than 100 adults/ha (Fig 1A). Medium movement speed scenarios provided better fits to mating activity observations by Stoner et al. [34] as compared to those from Delgado & Glazer [38], whereas low movement speed scenarios provided better fits to observations by Delgado & Glazer [38] (Fig 1A). All movement speed scenarios provided reasonable fits to empirical observations of spawning activity at densities of 100 or more adults/ha (Fig 2A).

Increased interbreeding rest periods led to reduced mating (Fig 1B) but did not impact spawning activity (Fig 2B). At low movement speeds, there were not substantial differences between simulation model predictions of mating at a given density with interbreeding rest periods between 0 to 2 days (Fig 1B). Shorter interbreeding rest periods provided better fits to empirical observations of mating activity by Stoner & Ray-Culp [32] and Stoner et al. [34]; longer interbreeding rest periods provided better fits to empirical observations of mating activity by Delgado & Glazer [38] (Fig 1B). At low densities, scent tracking increased mating success (e.g., 600% higher at 10 adults/ha); at high densities, it slightly reduced mating success (e.g., 18% lower at 2500 adults/ha; Fig 1C). Scent tracking had a negligible impact on spawning activity (Fig 2C). Increased conspecific perception distances led to increased mating (Fig 1D) and increased spawning (Fig 2D), especially at low densities. Increasing requirements for prior interactions to promote sexual facilitation of mating led to reduced mating (Fig 1E) and spawning (Fig 2E), particularly at low densities. Increasing mating success following ≥10 prior interactions (i.e., τ = 10, 25, or 50) more closely matched empirical observations at densities of <100 adults/ha (Figs 1E and 2E). A barrier to movement had minimal impact upon mating or spawning rates (Figs 1F and 2F). In general, simulations overestimated mating activity relative to observations by Delgado & Glazer [38] and did not account for their repeated observations of mating and spawning failure at high densities (Figs 1 and 2).

**Fig 3:**
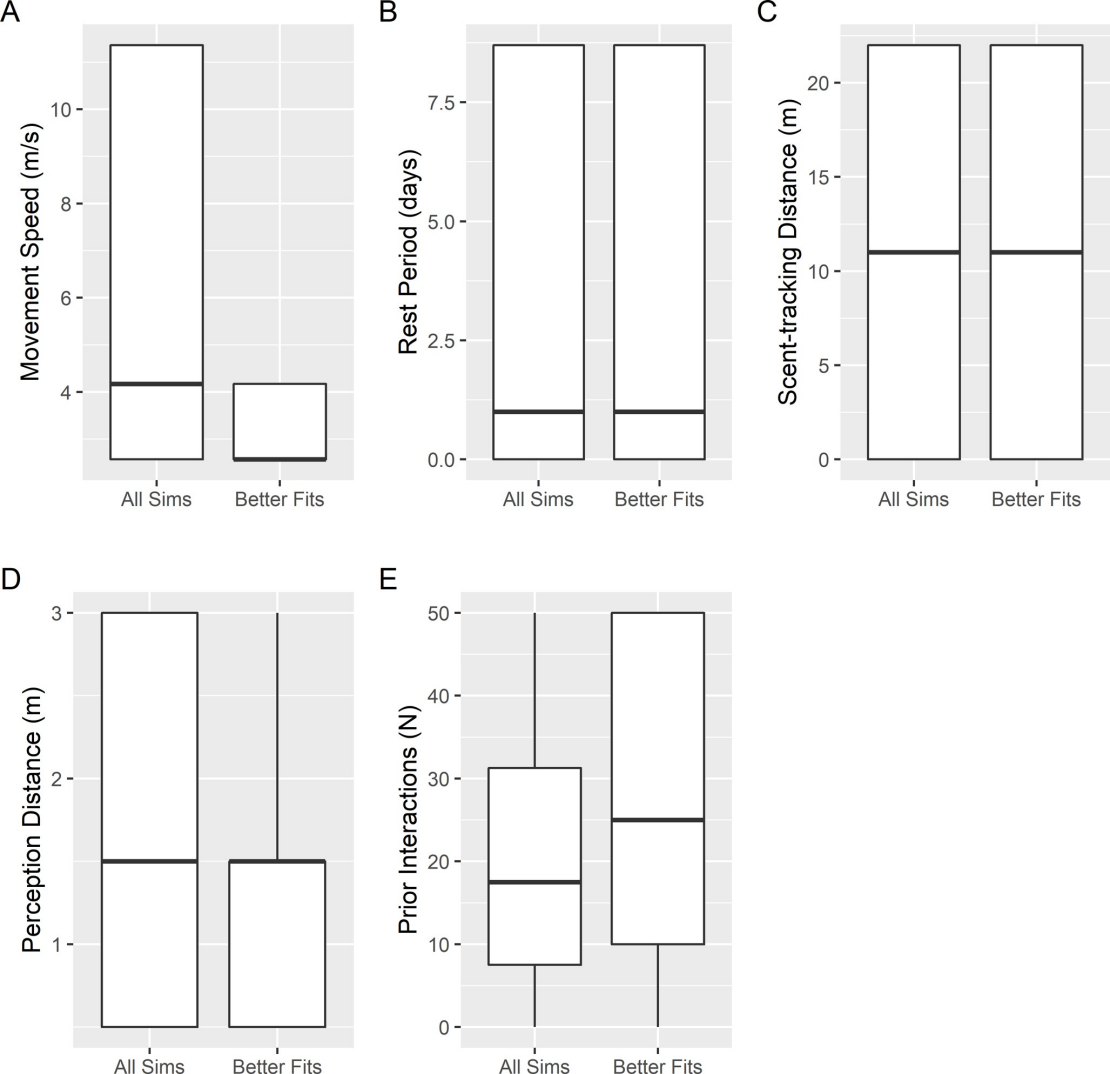
Simulation parameters resulting in superior model fits to empirical observations. Boxplots showing distribution of mechanistic simulation parameters across all simulations compared to distribution of parameters for simulations resulting in superior fits to empirical observations by Stoner & Ray-Culp [32] and Delgado & Glazer [38] as compared to logistic regression dose-response functions fit to the same data.

Of the 432 unique mechanistic simulations at 10 different densities of adult conch, 31% provided superior fits to empirical observations of percent mating by Stoner & Ray-Culp [32] and Stoner et al. [34] as compared to logistic regression functions fit to the same data (Table 2). The same was true for 9% of simulations compared to empirical observations of percent mating by Delgado & Glazer [38]. The model did not predict the high numbers of spawning observations with no corresponding mating activity observed by Delgado & Glazer [38]. When restricting comparisons to logistic functions fit to empirical data with either <100 adults/ha or at least one observed spawning event, 40% and 19% of simulations provided superior fits to empirical observations by Stoner & Ray-Culp [32] and Stoner et al. [34], and Delgado & Glazer [38], respectively (Table 3). All cases of superior fits were realized at low to medium movement velocities, limited perception distances, and relatively high prior interactions (e.g., ≥10; Fig 3). Rest period and scent tracking did not play a significant role in improving simulation model fits to empirical data (Fig 3).

**Table 2:** Dose-response logistic regression model fit statistics.

A) Stoner & Ray-Culp [32] and Stoner et al. [34]				
Parameter	Estimate	Std. Error	t-value	p-value
B	-56.18	98.10	-0.57	0.57
D	10.16	0.67	15.22	<2e-16
E	64.35	1.89	34.01	<2e-16
B) Delgado & Glazer [38]				
Parameter	Estimate	Std. Error	t-value	p-value
B	-71.04	120.03	-0.59	0.55
D	10.43	0.65	15.99	<2e-16
E	204.83	7.10	28.84	<2e-16

Parameter estimates for dose-response functions (Log-logistic with ED50 as parameter, with lower limit at 0) fit to censored (non-mating records at densities >100 adults/ha excluded) empirical observations of conch mating from A) Stoner & Ray-Culp [32] and Stoner et al. [34], and B) Delgado & Glazer [38].

Residual standard error: 5.814301 (124 degrees of freedom)

Residual standard error: 7.300855 (134 degrees of freedom)

**Table 3:** Best-fitting models.

	Boot ID	Velocity (m/s)	Rest Period (d)	Scent Tracking	Perception Distance (m)	Prior Interactions	Barrier	SSR Stoner	RMSE Stoner	SSR Glazer	RMSE Glazer
Stoner & Ray-Culp [32] and Stoner et al. [34]	419	4.17	8.7	0	3	25	one	5725	6.71	11150	9.02
	215	4.17	8.7	1	3	25	none	5794	6.75	10729	8.85
	431	4.17	8.7	1	3	25	one	5796	6.76	11123	9.01
	203	4.17	8.7	0	3	25	none	5809	6.76	10770	8.87
	346	2.57	8.7	0	3	10	one	5873	6.80	12525	9.56
	358	2.57	8.7	1	3	10	one	6012	6.88	12951	9.72
	130	2.57	8.7	0	3	10	none	6049	6.90	11806	9.28
	426	4.17	8.7	1	1.5	10	one	6109	6.94	12173	9.43
	432	4.17	8.7	1	3	50	one	6127	6.95	8980	8.10
	414	4.17	8.7	0	1.5	10	one	6142	6.95	11720	9.25
Glazer & Delgado [38]	200	4.17	8.7	0	1.5	50	none	7336	7.60	9044	8.12
	428	4.17	8.7	1	1.5	50	one	7528	7.70	9116	8.16
	348	2.57	8.7	0	3	50	one	7117	7.49	9120	8.16
	360	2.57	8.7	1	3	50	one	7359	7.61	9157	8.18
	144	2.57	8.7	1	3	50	none	7304	7.58	9191	8.19
	212	4.17	8.7	1	1.5	50	none	7414	7.64	9198	8.19
	132	2.57	8.7	0	3	50	none	7297	7.58	9214	8.20
	416	4.17	8.7	0	1.5	50	one	7527	7.70	9282	8.23
	342	2.57	8.7	0	1.5	10	one	7464	7.67	9519	8.34
	126	2.57	8.7	0	1.5	10	none	7725	7.80	9697	8.41

Parameters and goodness of fit statistics for the ten simulation models providing the best fits to empirical observations from Stoner & Ray-Culp [32] and Stoner et al. [34], and Delgado & Glazer [38] (with non-mating records at densities >100 adults/ha excluded).

**Fig 4:**
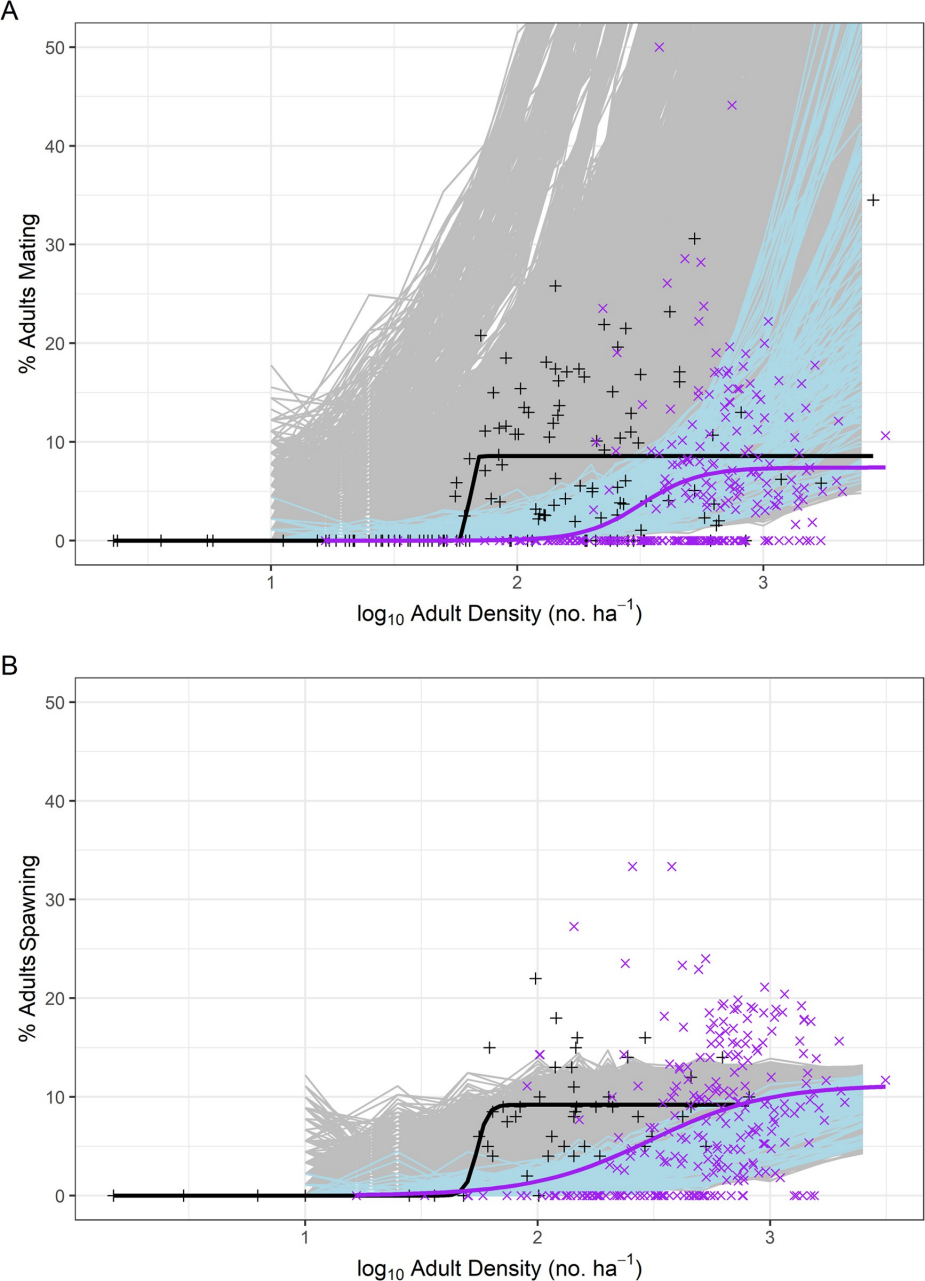
Simulated reproductive events relative to empirical observations. Simulation model outputs for percent of simulated adult queen conch successfully A) mating and B) spawning, relative to log_10_ adult density (No./ha) for all simulations (gray) and simulations providing superior fits (when non-mating activity above 100 adults/ha were excluded) to empirical data than logistic dose-response functions [54] fit to empirical observations by Stoner & Ray-Culp [32] and Stoner et al. [34] (black line and black crosses) and Delgado & Glazer [38] (purple line and purple x’s).

With the exception of a few observations of no mating activity at high adult densities from Delgado & Glazer [38], mechanistic simulations covered the range of empirically-observed mating activity by Stoner & Ray-Culp [32], Stoner et al. [34], and Delgado & Glazer [38] (Fig 4A). At very high adult densities (≥2000 adults/ha) simulations generally predicted higher rates of mating activity than were observed in the field (Fig 4A). Simulations providing superior fits to censored empirical data (observations of no mating activity at densities >100 adults/ha excluded) (blue lines in Fig 4A) tended to be more conservative in estimates of the percentage of mating activity, especially at low densities. Only simulations with low movement velocities, long rest periods, no scent tracking, and high requirements for prior interactions (≥10) were able to replicate observed trends for the lack of mating activity observed at low densities. Simulations generally underestimated the upper bound of spawning activity, with better-fitting simulation runs tending to be more conservative in estimates of spawning activity at low densities (Fig 4B).

**Fig 5:**
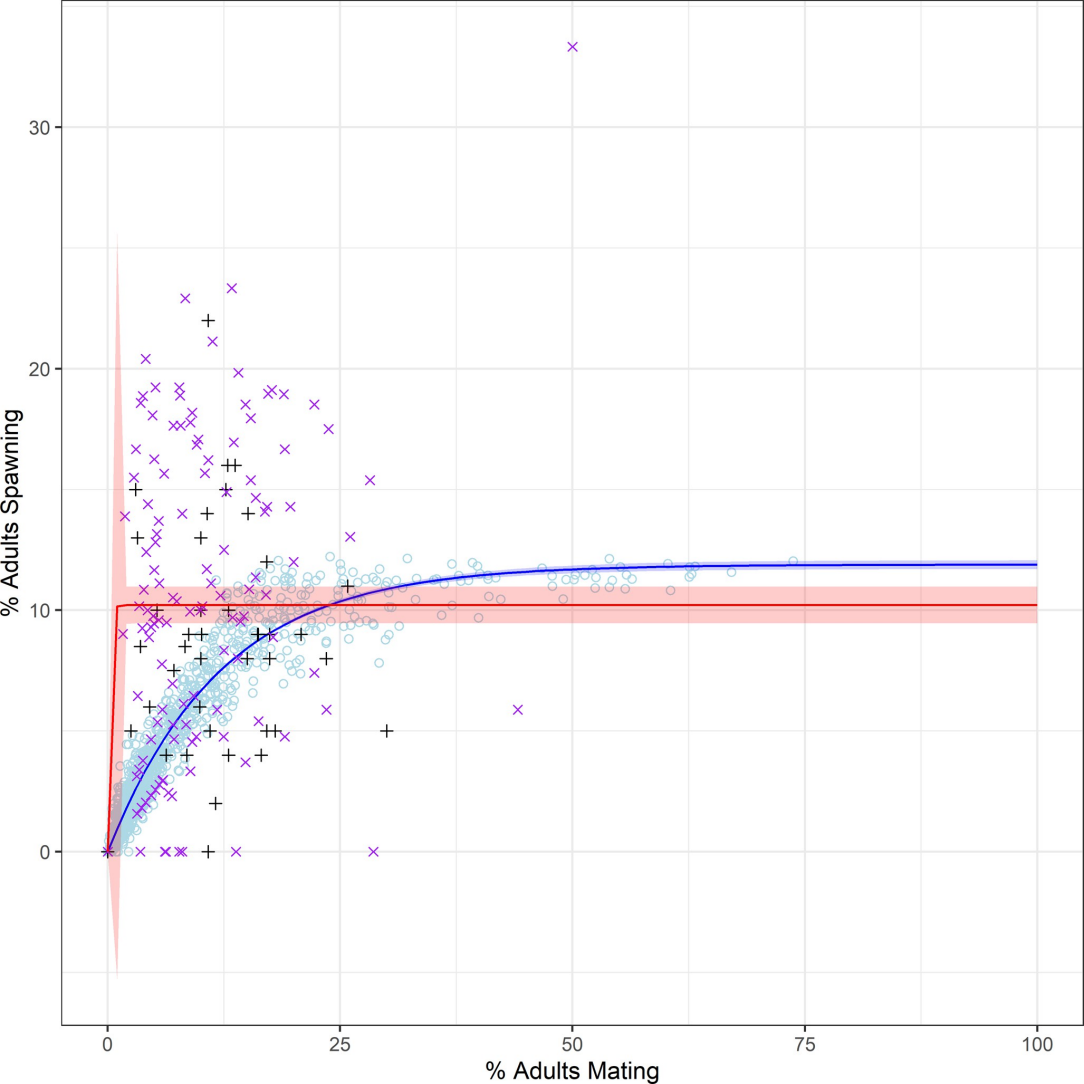
Mating and spawning activity. Relationship between mating and spawning activity across model simulations providing superior fits to empirical data (line and 95% confidence band in red) with logistic dose-response function fit (blue line) and 95% confidence band (blue), relative to empirical observations by Stoner & Ray-Culp [32] (black crosses) and Delgado & Glazer [38] (purple x’s).

Asymptotic regression functions with lower limit at 0 fit to simulation models using the ‘drc’ package in R [55] fit to the relationship between percent of adults mating and percent of adults spawning for simulation models; providing superior fits to empirical data showed an inflection point at around 25% mating (Fig 5: blue line). Simulations effectively captured mean trends in this relationship but underestimated the variability in spawning rates relative to the percent of adults mating when compared to censored (excluding observations of non-zero spawning at zero mating) combined empirical observations [32, 38]. Asymptotic regression functions with lower limit at 0 fit to censored empirical observations similarly underestimated overall variability in this relationship (Fig 5: red line; [32, 38]). Model fits for both functions are provided in Table 4.

**Table 4:** Asymptotic regression model fit statistics for mating vs. spawning.

A) Stoner & Ray-Culp [32] and Delgado & Glazer [38]				
Parameter	Estimate	Std. Error	t-value	p-value
D	10.22	0.38	26.57	<2e-16
E	0.19	4.97	0.04	0.97
B) Best-Fitting Simulations				
Parameter	Estimate	Std. Error	t-value	p-value
D	11.89	0.09	127.50	<2e-16
E	12.15	0.18	68.15	<2e-16

Parameter estimates for asymptotic regression, with lower limit at 0 fit to censored (>0% spawning at 0% mating excluded) combined empirical observations of conch mating from A) Stoner & Ray-Culp [32] and Delgado & Glazer [38] and B) data generated from best-fitting simulations.

Residual standard error: 4.97 (244 degrees of freedom)

Residual standard error: 0.71 (1718 degrees of freedom)

## Discussion

Depensatory mechanisms have been postulated as a major factor limiting the recovery of overharvested queen conch populations [34, 35]. Reproductive potential is primarily reduced by the removal of spawners from the population [35, 57]. Reproductive potential is secondarily limited by reduced densities, which increases the search time required for encountering potential mates [35]. Our simulations confirm this is especially limiting for slow-moving conch which require internal fertilization for successful mating. This limitation translates directly into limited recovery because the “search time” cost depletes both energy and time resources, meaning gametogenesis will not proceed at maximal rates and thus, populations will not reproduce to their full capacity. Our simulations confirm that limitation on mate finding associated with density is the primary driver behind observed patterns in mating and spawning activity, but similar to field observations by Gascoigne & Lipcius [58], challenges associated with mate finding cannot be the only explanation for lack of reproductive activity at low densities.

Our simulations also indicate that high movement speeds and extensive scent tracking are unlikely explanations for observed trends in queen conch mating and spawning activity (Figs 1A, 1C, 2A and 2C). Simulations of these factors provided poor fits to empirical data and increased levels of movement or taxis pushed simulations further from observed trends. Additionally, simulations suggested a barrier to movement associated with microhabitat features has little impact on the percentage of the population mating or spawning, assuming the population is uniformly distributed in space (Figs 1F and 2F). This is likely because in simulations, the barrier serves to reflect adult conch back at other conspecifics rather than slowing their rate of movement. This could be a cause for concern with regard to genetic diversity by increasing mating interactions between the same individuals, but does not appear to reduce mating activity. Macroscale barriers preventing movements between deep-water and shallow-water habitats may directly impact reproductive output; however, to be consistent at the scale commonly used to report conch densities [34, 38] our simulations were intended only to represent density at a one-hectare spawning aggregation site.

Although interbreeding rest period impacted mating activity in simulations, it did not have a corresponding impact on spawning (Figs 1B and 2B). This is because the longest interbreeding rest period was parameterized according to the interspawning period defined for female conch from Weil & Laughlin [11]. When evaluated independently, the best mechanistic explanation for the reduced mating and spawning activity at all densities observed by Delgado & Glazer [38] relative to Stoner & Ray-Culp [32] and Stoner et al. [34] was reduced conspecific perception distance required for successful mate finding (Figs 1D and 2D). The only mechanistic explanation for the absence of empirical observations of mating or spawning activity at low densities was a relatively high requirement (τ ≥ 10) for prior conspecific interactions attributed to sexual facilitation (Figs 1E and 2E); however, low movement velocities and long rest periods were also necessary to replicate field observations (Fig 4; Table 2). Although simulations were able to replicate the range of empirical observations for percentage of the population mating (Fig 4), the variability in empirical observations suggests that no single simulation run can perfectly capture the reproductive dynamics of queen conch. There is a likely interaction between the various mechanistic and environmental factors within the specific sampling environments that plays a significant role in the percentage of mating observed. Aside from the potential influence of water clarity on perception distance and the role that microhabitat features may play in inhibiting direct contact (discussed later in this section), behavioral shifts associated with unrelated daily activities (i.e., foraging, migration, burying, changing movement speeds, and responding to external stimuli) could also be associated with the disconnect between simulated and empirical observations. Such minor changes in behavior would likely decrease mating frequency and/or make it difficult for observers to accurately capture mating activity during field surveys.

Simulations generally overestimated mating at low densities with the exception of runs that required sexual facilitation, low movement velocities, and longer rest periods. Sexual facilitation was implemented as an increasing probability of mating success with increasing conspecific encounters; however, empirical data and simulation results both suggest implementation as a knife-edged requirement might improve model fitting and should be explored in future iterations. Similarly, simulations tended to underestimate conch spawning activity relative to empirical observations, suggesting that the delay in egg deposition (8.7 ± 4.9 d [11]) may have been too conservative, particularly since female queen conch are capable of storing sperm for approximately one month prior to spawning [11].

Queen conch move by anchoring the sickle-shaped operculum against the seafloor and thrusting the foot backward, propelling the shell forward a half-body length at a time [1]. Adults move at varying speeds throughout the year and are capable of extensive seasonal migrations to and from historic spawning grounds. As adults migrate to these spawning areas aggregations are typically formed and individual movements slow when mating and spawning activities begin [5]. Conch may move shorter distances as needed to forage or actively locate compatible mates, but typically remain within smaller areas until returning to their feeding grounds at the conclusion of the reproductive season [5, 16]. Our model simulations using low (2.5 m/d) and medium (4.17 m/d) within-aggregation movement speeds more closely followed field observations of mating activity compared to high (11.36 m/d) movement speeds. The high movement speed used from Doerr and Hill [15] was compiled from an extensive tracking period and included combinations of fine-scale daily movements and large-scale reproductive migrations; however, it did not include estimates of within-aggregation movement rates. At higher densities within an aggregation, individuals would not need to travel far to locate a receptive mate. Likewise, easy access to sufficient food supply within the aggregation area would eliminate the need for short foraging trips and allow conch to continue to mate and spawn at high frequencies. Reducing movements to minimize excess energy expenditures during mating and spawning would help to ensure maximum reproductive output, particularly for females, in the form of high overall egg production.

Simulations suggested that longer interbreeding rest periods could play an important limiting role in reproductive success. It is possible that female receptivity to mates will be lower during oogenesis. In high-density aggregations, mating concurrent with egg laying is not uncommon [32]; however, after spawning, females might not attract mates or might avoid mates, creating a rest period. Delays or rest periods in female receptivity are probably not explained by a bioenergetic need to forage after a mating event, as conch have been observed foraging while mating [32, 59]. However, the developmental period between egg masses may have a bioenergetic link, as the development and deposition of large egg masses is an energetically costly event requiring either substantial body reserves or additional energy intake through foraging to be repeated [11, 60]. The bioenergetic recovery time needed between spawning events would likely increase if overfishing of conch on productive shallow-water habitats were to drive the population to deeper, less productive habitats, further decreasing overall reproductive output [8, 61].

Because queen conch do not move using a slime trail, scent-trail following would presumably be limited in spatiotemporal scope. Our simulations suggested that one-day duration scent tracking out to near the maximum daily movement distance would greatly increase mating activity at lower densities. Ecologically, this could be explained as an increased efficiency in mate finding offsetting the slow movement speed of reproductive adults. Our simulations also suggested that scent tracking at higher densities might actually lead to a slight reduction in mating activity. This result could be explained by the inability to focus on and track a single individual, leading to inefficiencies in the movement path. This is similar to the well documented “confusion effect” for predatory fish targeting individuals within large schools of fish [62].

In addition to the direct removal of spawners and the increased mate encounter times caused by lower densities, a third potential depensatory mechanism is the breakdown of a positive feedback loop between contact with males and the rate of gametogenesis and spawning in females [35, 48–50]. When reproductive fitness declines such that per capita population growth rate becomes negative, localized extinction may result [63, 64]. This sexual facilitation could be accomplished through direct contact or chemical cues [35]. Copulation in conch is more likely to occur in spawning than non-spawning females, providing an additional positive feedback mechanism that amplifies the effect at high densities [65]. Our model provides mechanistic confirmation that the reductions of densities caused by overharvesting of spawning aggregations increases the probability of recruitment failure beyond what would be anticipated from delays in mate finding alone. This is consistent with field experiment findings from Gascoigne & Lipcius [58], which indicate that in addition to depensatory mechanisms associated with mate finding, delayed functional maturity at low-density sites can explain declines in reproductive activity. As such, understanding depensatory thresholds seems absolutely critical to effective fisheries management for the species [38].

Adult density had the largest effect on mating and spawning activity. Sexual facilitation was a necessary mechanistic explanation to replicate extremely low (or lack of) mating rates observed at low densities. Only runs requiring higher levels of sexual facilitation replicated patterns reported in the field with apparent thresholds below which no mating was observed [34, 38]. Similarly, perception distance was a major controlling factor of mating and spawning rates at higher densities. When viewed independently of other factors and queen conch were assumed to have very limited (max of 0.5 m) perception distance for mating encounters, simulation outputs more closely patterned empirical observations in the Florida Keys back reef by Delgado & Glazer [38] (Fig 2D). When viewed independently of other factors and queen conch were assumed to have fairly broad (up to 3 m) perception distance, simulation outputs more closely patterned empirical observations in Bahamian waters by Stoner & Ray-Culp [32] and Stoner et al. [34]. Ecologically, perception distance could be interpreted as near-field ability to visually or chemically locate potential mates. In the field, perception distance might vary based on the strength and duration of chemical cues in the water or on the substrate, the direction and strength of current flows between potential mates, and water clarity. Inferring from studies with other gastropods, queen conch likely detect conspecifics and predators through their chemosensitive tentacles and use their keen eyesight to orient subsequent movements [66]. The eyes of *Strombus/Lobatus* are among the best developed of those found in gastropods [67], and it is likely that conch can converge on objects during visual fixation [68]. We are unaware of any studies of how far queen conch can see, but our simulations suggest some of the differences in mating activity observed between Stoner et al. [34] and Delgado & Glazer [38] could be attributed to differences in perception distance. It is possible that the clear waters and relatively flat, shallow habitats of the Bahamas provide a greater perception distance (closer to 3 m) than the rugose, lower-visibility back-reef sites surveyed by Delgado & Glazer [38]. Further studies on the conspecific perception distance and visual acuity of queen conch are needed to validate this hypothesis.

Conch can be confined by ecological barriers such as fragmented habitats, the presence of extensive bare sand plains that lack food resources, or areas that may expose them to potential desiccation or anoxic conditions [69, 70]. Natural barriers to movement can serve to isolate populations through suppressed immigration of juvenile and adult conch. For example, in the Florida Keys, the East Harbour Lobster and Conch Reserve in South Caicos, and certain areas of Lee Stocking Island, Bahamas, conch are separated from surrounding habitats by coral ledges, sand bars, and offshore reefs, respectively [5, 59, 69]. In simulations, because conch were uniformly distributed, the single barrier reduced interactions with distant individuals, but this limitation on dispersal was offset by increased interactions with nearby individuals. In the wild, this type of environmental bottleneck might cause concerns for genetic diversity within the population [71], although many factors would come into play that were not evaluated in our simulations.

Our simulations assumed conch were reproductively active during 10 days of the peak spawning period. The range of model simulations encompassed the range of variation in empirical observations of percent mating at given densities (see Fig 4). In reality, a combination of mechanistic and environmental stochasticity likely explains the variance observed in the field. In marine species, including mollusks, environmental triggers including rapid changes in water temperature or the detection of conspecific gametes in the water are often implicated in the initiation of gametogenesis and reproductive activities [72]. For queen conch, multiple studies have identified increasing water temperature and photoperiod as the stimulus for reproductive migrations and the subsequent initiation of mating [5, 11], and recent evidence has verified the presence of sex hormones in conch feces [73]. Concentrations of estrogen, progesterone, and testosterone increase in conjunction with each phase of the conch reproductive season, indicating that these hormones are linked to the reproductive process [73]. Active hormone detection by conspecifics would positively influence encounter rates of low-density populations and could explain our model outcomes for scent tracking simulations where mating success increased at lower densities.

Increasing water temperature due to climate change is likely to alter the timing and duration of the queen conch reproductive season. In warmer regions, conch have been observed mating and spawning year round [1, 74]; however, reproduction can also cease as temperatures approach 31°C [75]. Increasing water temperatures may initially extend the reproductive season and shift peak mating and spawning periods, but further increases may subsequently shorten the season as temperatures reach a threshold. If adult conch respond to temperature increases by moving from shallow mating grounds to deeper waters with potentially diminished habitat quality, overall reproductive output may decrease.

In addition to environmental drivers that influence reproductive success, there are biological factors that can negatively impact individual reproductive output. Histological examination of digestive gland samples collected from queen conch throughout the majority of their Caribbean range revealed the presence of a coccidian Apicomplexan inclusion body [76]. A higher abundance of these inclusion bodies appeared to correspond with reproductive abnormalities such as reduced frequency of gametogenesis, delayed maturity, and low gonad activity and spawn stages [77, 78]. However, recent studies have suggested that these inclusion bodies are not parasitic and the individuals sampled appeared to be reproductively healthy [79, 80]. This recent finding warrants further research into overall reproductive impacts, particularly for adult conch at reduced density levels. This could be useful in refining our model to further examine reproductive impacts in populations at seemingly adequate adult densities but exhibiting reduced overall spawning activity.

This model may also be useful for identifying reference points that avoid recruitment failure. Cross-shelf density thresholds for mating and spawning reported by Stoner & Ray-Culp [32] in the Bahamas were 56 adults/ha for mating and 48 adults/ha for spawning. Similarly, Stoner et al. [34] report threshold densities for mating of 47 to 74 adults/ha. By contrast, Delgado & Glazer [38] report aggregation density thresholds of 204 adults/ha for mating and 90 adults/ha for spawning, respectively. These discrepancies may be partially explained by differences in geographic location and survey methodology. Previous studies of queen conch in nearshore areas of Florida have reported a complete lack of reproductive activity with individuals demonstrating reduced gonadal development [81, 82]. Though the offshore aggregation surveys conducted by Delgado & Glazer [38] may not have included any of these inhibited nearshore adults, the presence of reproductively inactive conch in the region reduces the probability of reproductively active individuals successfully locating mates. Additionally, intra-aggregation densities are necessarily higher than cross-shelf densities recorded outside the spawning season, as they draw individuals from across the spawning catchment area. Stoner & Ray-Culp [32] recorded reproductive behaviors for conch outside the survey circles in which they estimated density and individuals were counted as mating if they were in mating position but not actually copulating [38]. These differing methodological approaches likely explain some of the disparities between our simulation results and minimum reproductive threshold densities observed in the field.

The United Nations Environment Programme (UNEP) has recommended a reference point of 100 adults/ha to avoid impacts to recruitment [83]. Recent studies [38] and practical application in Jamaica [84] have suggested this threshold may be insufficient to avoid population collapse. Our simulations of mating dynamics within spawning aggregations suggested an inflection point with approximately 25% of the population mating resulting in near-peak reproductive potential, as measured by percent spawning (Fig 5). We could not replicate the variability in the relationship between percent mating and percent spawning observed in field studies (Fig 5; [32, 38]), although the mean relationship between these reproductive events was well captured by the simulations. The wide temporal disconnect between mating and an observed spawning event is further complicated by the ability of female conch to store sperm for extended periods of time [11] and the inability to observe potential spawning females for extended periods of time following mating. Possibly as a result, simulations incorporating sexual facilitation (Figs 1E and 2E) suggest aggregation densities >200 adults/ha are necessary to achieve high levels of spawning output. Furthermore, the best-fitting simulations suggest that significant increases in spawning begin accruing at densities >250 adults/ha (Fig 4B).

For queen conch and similar motile invertebrates that must locate conspecifics for reproduction, population density is one of the most critical factors in maintaining the reproductive output of the stock. However, other drivers of mating and spawning success examined in our model simulations indicate that population density is not the only factor to consider. Our simulation results suggest that biological characteristics of queen conch such as scent-tracking ability, rest periods between mating events, sexual facilitation, perception distance, barriers to movement, and movement speeds interact with population density to varying degrees to influence mating and spawning frequencies, and thus, total reproductive output. Further modelling exercises incorporating refined biological parameters or additional environmental drivers can potentially be used to guide the development of innovative management strategies and enhance conservation efforts.

## Supporting information

S1 File R code for the queen conch reproduction simulations. (R)

S1 Fig Plot of barriers to movement. Barriers to movement (shaded polygons) relative to movements of male (+) and female (x) conch. Movement barriers were simulated to evaluate the impacts of microhabitat features on reproductive dynamics. Features ranged from single linear barriers to multiple complex barriers. A single large barrier (B) could be interpreted as a transition between habitats with minimal connectivity. Several long barriers (C) could be interpreted as several habitat transitions. Pylons (D) and horseshoes (F) could be interpreted as many small- to medium-sized natural or artificial barriers to movement (e.g., bridge pylons, artificial reefs, or patch reefs). A cross (E) could be interpreted as a single large barrier to movement such as a high relief coral reef.
(TIF)
