# Supplementary figures and images for: Limiting factors for queen conch (Lobatus gigas) reproduction: A simulation-based evaluation
Source: PLoS One. 2022 Mar 9;17(3):e0251219. doi: 10.1371/journal.pone.0251219 (PMC8906866; doi:10.1371/journal.pone.0251219)

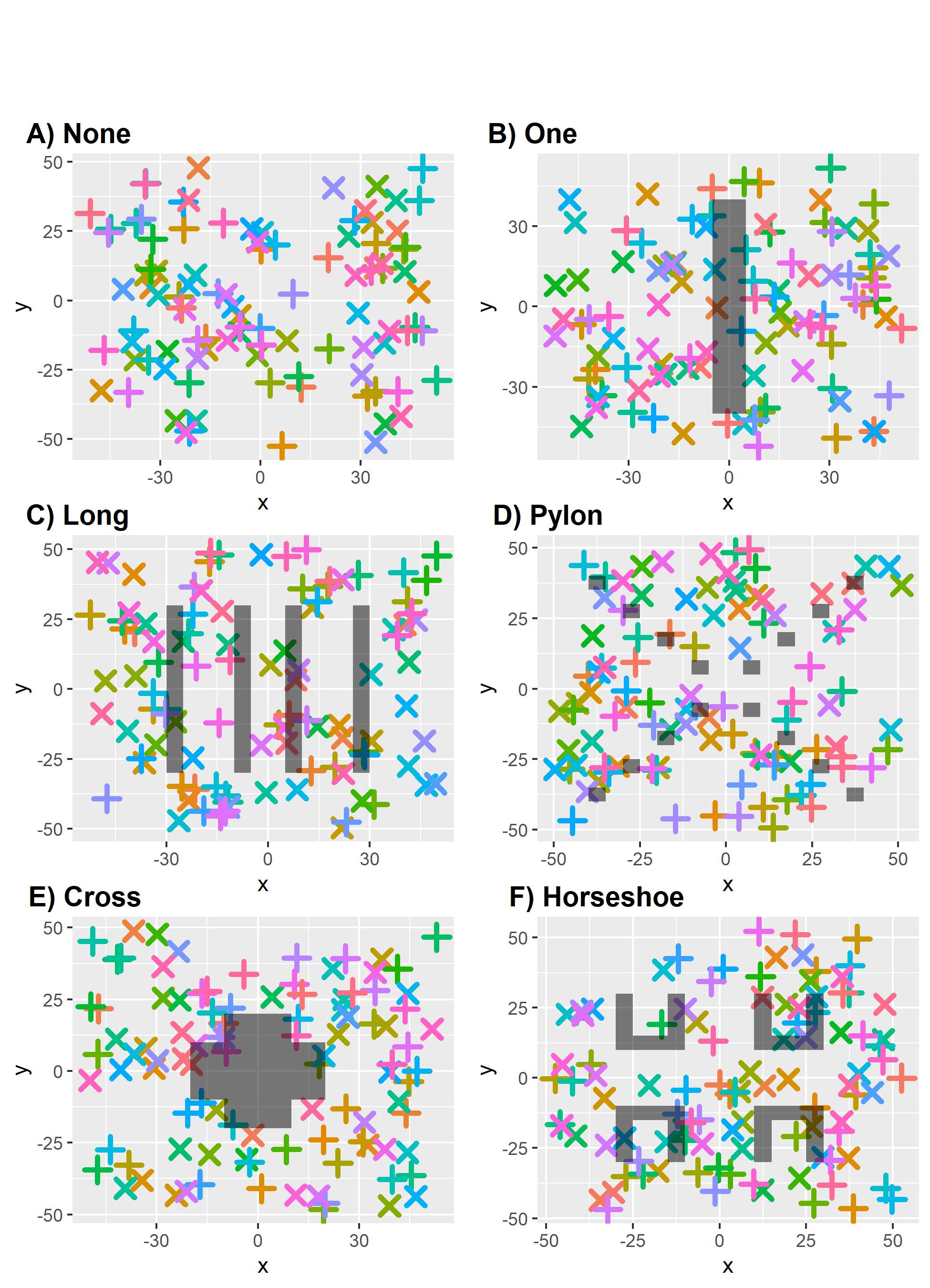

Supplement: S1 Fig — Barriers to movement (shaded polygons) relative to movements of male (+) and female (x) conch. Movement barriers were simulated to evaluate the impacts of microhabitat features on reproductive dynamics. Features ranged from single linear barriers to multiple complex barriers. A single large barrier (B) could be interpreted as a transition between habitats with minimal connectivity. Several long barriers (C) could be interpreted as several habitat transitions. Pylons (D) and horseshoes (F) could be interpreted as many small- to medium-sized natural or artificial barriers to movement (e.g., bridge pylons, artificial reefs, or patch reefs). A cross (E) could be interpreted as a single large barrier to movement such as a high relief coral reef. (TIF) [file pone.0251219.s002.tif]
